# Client and facility level determinants of quality of care in family planning services in Ethiopia: Multilevel modelling

**DOI:** 10.1371/journal.pone.0179167

**Published:** 2017-06-16

**Authors:** Gizachew Assefa Tessema, Mohammad Afzal Mahmood, Judith Streak Gomersall, Yibeltal Assefa, Theodros Getachew Zemedu, Mengistu Kifle, Caroline O. Laurence

**Affiliations:** 1 School of Public Health, The University of Adelaide, Adelaide, Australia; 2 Department of Reproductive Health, Institute of Public Health, University of Gondar, Gondar, Ethiopia; 3 School of Public Health, University of Queensland, Brisbane, Australia; 4 Health System and Reproductive Health Research Directorate, Ethiopian Public Health Institute, Addis Ababa, Ethiopia; 5 Federal Ministry of Health, Addis Ababa, Ethiopia; University of West London, UNITED KINGDOM

## Abstract

**Introduction:**

Over the last two decades, while contraceptive use has improved in Ethiopia, the contraceptive prevalence rate remains low. In addition to socio-demographic and cultural factors, the quality of care in Family Planning (FP) services is an important determining factor of FP utilization. However, little research exists on the determinants of quality of care in FP services in Ethiopia. This study aims to identify the client and facility level determinants of quality of care in FP services in Ethiopia.

**Methods:**

This study was based on the first Ethiopian Services Provision Assessment Plus (ESPA+) survey conducted in 2014. A total of 1247 clients nested in 374 health facilities were included in the analysis. Multilevel mixed-effects logistic regression modelling was conducted. The outcome variable, client satisfaction, was created using polychoric principal component analysis using eleven facets that reflect client satisfaction.

**Results:**

The results showed that both client-level and facility-level factors were associated with quality of care in FP services in Ethiopia. At the client-level; provision of information on potential side effects of contraceptive method (AOR = 5.22, 95% CI: 2.13–12.80), and number of history and physical assessments (AOR = 1.19, 95% CI: 1.03–1.34) were positively associated with client satisfaction, whereas waiting times of 30 minutes to two hours (AOR = 0.11, 95% CI: 0.03–0.33) was negatively associated with client satisfaction. At the facility-level; urban location (AOR = 4.61, 95% CI: 1.04–20.58), and availability of FP guidelines/protocols for providers (AOR = 4.90, 95% CI: 1.19–20.19) had positive significant effect on client satisfaction.

**Conclusion:**

Quality improvement programs in FP services in Ethiopia should focus on shortening waiting times and provision of information about the potential side effects of contraceptive methods. It is also important to improve health providers’ skills in thorough client history taking and physical assessment. Further distribution and implementation of best practice guidelines for providers working in the FP services must be a priority.

## Introduction

In 2015, the World Health Organization (WHO) estimated that 303,000 women were dying globally from pregnancy-related causes and complications, of which 66% occurred in sub-Saharan Africa. Ethiopia is ranking fourth in the top ten countries that accounted for 59% of the maternal deaths globally [1]. According to the recent Ethiopian Demographic and Health Survey (EDHS), the maternal mortality ratio was 412 per 100,000 live births in 2016 [2].

In response to the high rate of maternal deaths in developing countries, in 1996 the WHO identified Family Planning (FP) services as one of the key strategies of the safe motherhood initiative [3] aimed to reduced maternal deaths. This followed the outcome of the 1994 International Conference on Population and Development (ICPD) where FP was seen as important in meeting reproductive health needs for couples and families [4]. In 2012, analysis of data from 172 countries showed that contraceptive use had averted 44% of maternal deaths [5]. In addition, evidence points to family planning’s key role in also reducing infant mortality. For example, a study focusing on developing countries found that it reduced infant mortality by 10%, and childhood mortality by 21% [6]. The contribution of FP in reducing maternal mortality is due to its role in preventing unintended pregnancy and complications such as abortions and preventing teens pregnancy [7,8]. Moreover, the adequate interval between successive pregnancies gained from use of FP helps to prevent preterm, low birth weight, and small-for-gestational age infants, factors associated with early child mortality [9,10].

In Ethiopia, little was known about FP before the mid-1960s. Modern FP was introduced in Ethiopia half a century ago by the Family Guidance Association of Ethiopia (FGAE). Initially, the FGAE provided services in a single room by one nurse, at St Peter Hospital in Addis Ababa. Later, the Ministry of Health (MOH) enhanced the effort through provision of FP services as part of the Maternal and Child Health (MCH) services in all levels of health facilities [11,12]. In 1996, the MOH developed guidelines for FP services to expand and ensure the quality of care in FP and these were revised in 2011. In the latest guidelines, the MOH outlined that FP services should be delivered through community-based, facility-based, and outreach modalities [11]. These developments have contributed to significant improvements in the Contraceptive Prevalence Rate (CPR) in the past 16 years (8% in 2000, 14.7% in 2005, 28.6% in 2011, and 36% in 2016) [2,13–15]. Nevertheless, the fertility rate remains high in Ethiopia, with an average total fertility rate of 4.6 in 2016 [2].

Despite the improvements in FP use in Ethiopia, there are still areas for improvement. More than one third (37%) of Ethiopian women who commence contraception discontinue use within 12 months and the discontinuation rate varies by the method of contraception. According to the EDHS 2011 report, the discontinuation rate for oral contraceptive pills was 70%, 62% for male condoms, and 34% for injectable methods[15]. The reasons for such high discontinuation rates are not clearly understood. However, several studies from developing countries have found that poor quality of care in FP services is a prime reason for high rates of discontinuation, reduced utilization of FP services, non-compliance, and high unintended pregnancies [16–22]. A study conducted in Northern Ethiopia found that women who did not receive a follow-up appointment to the FP service or had not been satisfied by the service provided were more likely to discontinue their FP use[23]. In 2015, the Health Sector Transformation Plan (HSTP) was introduced in Ethiopia. One of the goals of the HSTP is to increase the CPR to 55% and reduce the unmet need for FP use (percentage of those who want to stop or delay childbearing but are not using any method of contraception) to 10% by 2020 [24]. Improving the quality of care in FP services may be one strategy to help achieve these goals.

Globally, assessments of quality of care in FP services have largely been informed by two frameworks: the Donabedian and the Bruce/Jain frameworks. These have identified a range of outcomes such as client satisfaction, change in contraceptive knowledge and behaviour, and reduction in fertility as important in measuring the quality of care in FP services [25,26]. However, in resource limited countries such as Ethiopia, most of the long term outcome measures have rarely been collected, with short term outcome measures such as client satisfaction more frequently collected as an indicator for quality of care.

To date only a few small-scale studies have been conducted to assess quality of care in FP services in Ethiopia [27–31]. These studies were limited to public health facilities [30,31] or failed in identifying the factors determining quality of care in FP services [27–29]. These studies that identified factors associated with quality of care in FP services were pointed to quality of care was associated with client’s age, educational status, providers’ experiences, client’s waiting time, clients' perception on adequacy of information during consultation, ease of getting the health facility [30,31]. A recent systematic review, that considered quantitative and qualitative studies, showed that few studies have assessed determinates of quality of care in FP services in Africa [32]. The first ever Ethiopian Services Provision Assessment Plus (ESPA+) survey, undertaken in 2014 [33]. This study was aimed to identify the client and facility-level determinants of quality of care in FP services in Ethiopia.

## Methods

### Study context

This study was conducted in Ethiopia which has nine administrative regions and two city administrations. The country had a population of 102 million (second largest in Africa) and a total fertility rate of 4.6 in 2016 [2,34]. In 2010/11, there were 202 hospitals, 3292 health centers, 15618 health posts, and 3990 private clinics (higher clinics, medium clinics, and lower clinics) providing various health services including FP [35].

### Study design

This study employed a secondary analysis of data obtained from ESPA+ 2014 survey. Three databases—namely, the facility inventory data for facilities providing FP services, the client-provider observation data, and clients exit interview data—were linked for this analysis.

### Overview of the Ethiopian Services Provision Assessment Plus (ESPA+) 2014

The ESPA+ 2014 survey provided a comprehensive picture of the quality and availability of a basic health services that includes maternal and child health, reproductive health services, and infectious diseases. Guided through the Services Provisions Assessment (SPA) methodology which was initially developed by Demographic and Health Survey (DHS) Program, the ESPA+ survey was conducted on a nationally representative sample of health facilities. Similar SPA surveys have been conducted in several African countries [36]. The ESPA+ 2014 survey comprised facility inventory assessment, client exit interviews, observation of client-provider, and health provider’s interviews. The survey was conducted by the Ethiopian Public Health Institute (EPHI) in collaboration with the Ministry of Health. ICF International provided technical assistance. The survey involved a representative sample of these health facilities of all levels (lower and higher) and types (public and private) [33]. Details of the ESPA+ instruments, data collection methods, recruitment and data quality assurance procedures is provided elsewhere [33].

### Sampling and sample size

**Fig 1:**
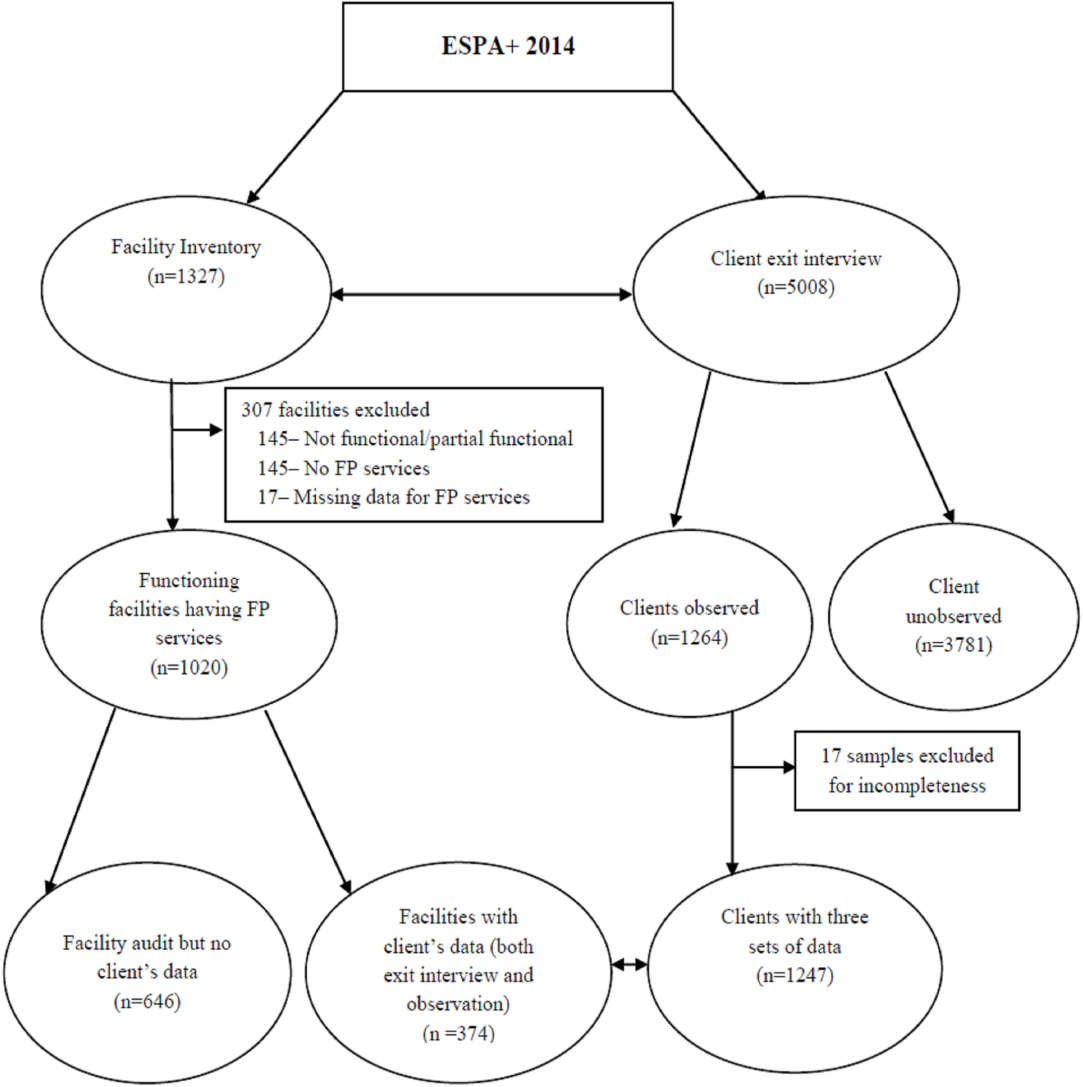
Schematic presentation of samples for identifying client and facility level determinants of quality of care in family planning services in Ethiopia.

A total of 1327 health facilities were visited during the survey, of which 1037 provided FP services. In these health facilities, 5008 clients were invited to undertake client exit interviews of which client-provider interaction observations were undertaken for 1264 clients in a subset of 374 health facilities. For analysis, 17 samples were excluded due to incompleteness of important variables related to the outcome measure. The final analysis was therefore based on 1247 clients nested in 374 facilities providing FP services **(Fig 1).**

### Variables

#### Outcome variable

The outcome variable of this study was *client satisfaction*. There are three advantages of using client satisfaction as the outcome measure. Firstly, client satisfaction is one of the key determinants of uptake and continued use of FP services [16,37]. Secondly, it signals other aspects of quality of care including structural and process aspects of quality of care in FP services [16,37,38]. Thirdly, client satisfaction reflects the perception of health care consumers (FP clients) on quality of care on existing health services.

Client satisfaction was measured using clients’ exit interview responses to questions about the health service quality, assessed by the problems encountered to clients during their visit to health facilities for FP services. In this regard, respondents were asked to rate eleven facets that reflect clients’ perceptions of the quality of the visit (Table 1). The responses were then aggregated into an index using polychoric principal component analysis for discrete variables [39]. Principal component analysis was undertaken using the correlation matrix of these eleven facets. The first principal component was used as the index for client satisfaction. As the aggregated index value was skewed, we created a dichotomised variable using the median score as a cutoff point. The median score for the aggregate value was 3.91. Finally, a binary outcome of client satisfaction was devised as more satisfied if a score was 3.91 or greater, and as less satisfied if a score was less than 3.91.

**Table 1:** Description of variables used in the analysis.

Variable	Type of variable and/or definitions	Sources of dataset
**Outcome variable**		
Client satisfaction	Binary variable (less satisfied Vs more satisfied) The variable created using polychoric principal component analysis based on 11 aspects that clients have been asked to report if they encountered a problem during visit to the facility. These aspects included:1) time clients waited to see provider; 2) ability to discuss problems or concerns about your method; 3) amount of explanation you received about the problem or treatment; 4) privacy from having others see the examination; 5) privacy from having others hear your consultation discussion; 6) availability of FP commodities at this facility; 7) the hours of service at this facility, i.e., when they open and close; 8) the number of days services are available to you; 9) the cleanliness of the facility; 10) how the staff treated clients; 11) cost for services or treatments	Client exit interview
**Independent variables**		
***Client characteristics***		
Sex	Binary (male, female)	Client exit interview
Age (in years)	Continuous	Client exit interview
Educational status	Categorical (no education, primary education, secondary education, tertiary education)	
Types of client	Binary (new, repeated); clients were regarded as new clients if it was their first time to seek FP services; otherwise regarded as repeated clients.	Client exit interview
***Process aspects / client-provider interaction***		
Information provided about how to use the contraceptive method	Binary (no, yes); whether or not the clients received explanation about how to use the contraceptive method	Client-provider observation
Information provided about contraceptive method’s potential methods side effects	Binary (no, yes); whether or not clients have been told about possible side effects of the contraceptive method	Client-provider observation
Information provided on what to do if problem occurs	Binary (no, yes); whether or not clients have been told what to do if they have any problems	Client-provider observation
Information provided on when to return for follow-up	Binary (no, yes); whether or not clients have been informed when to return for follow-up	Client-provider observation
Partner’s attitude towards FP asked	Binary (no, yes); whether or not clients asked about their partner attitude towards FP (in favour of, or against the idea of family planning)	Client-provider observation
Client’s number of sexual partners asked	Binary (no, yes); whether or not client’s number of sexual partner(s) asked by the provider	Client-provider observation
Client’s perceived risk of HIV/STI asked	Binary (no, yes); whether or not client’s perceived risk of HIV/STI asked by the provider	Client-provider observation
Client’s concerns about the methods addressed	Binary (no, yes); whether or not provider asked clients about any question or concerns about the current contraceptive method; or clients expressed concerns about the contraceptive method including possible side effects	Client-provider observation
Clients asked questions to the providers	Binary (no, yes); whether or not clients expressed concerns or asked questions about the contraceptive method including possible side effects	Client-provider observation
Information about condom use for STI prevention provided	Binary (no, yes); whether or not clients have been informed about condom use for STI prevention	Client-provider observation
Information about dual method use for HIV/STI prevention provided	Binary (no, yes); whether or not clients have been informed about use of dual contraceptive methods that included condom and one of hormonal contraceptive methods to prevent HIV/STI	Client-provider observation
Privacy maintained	Binary (no, yes); whether both auditory and visual privacy of clients maintained during consultation	Client-provider observation
Confidentiality assured	Binary (no, yes); whether the clients received verbal reassurance about their privacy	Client-provider observation
Duration of consultation in minutes	Continuous; Number of minutes provider spent for consultation	Client-provider observation
Number of history and physical assessment	Continuous (0–14); An index based on 14 aspects that the provider assessed or conducted for clients. The aspects involved if the provider asked clients about their last time of delivery, age, last time of mensuration, history of breastfeeding, history of regular menses, number of living children, desire for additional children, asked for smoking, asked for any chronic illness, asked for Sexually Transmitted Infection (STI) symptoms, took client’s blood pressure, and took client’s body weight.	Client-provider observation
Client’s waiting time	The total time in minutes elapsed between client’s arrival to the facility and the time when she has been seen by the provider for consultation on FP services. Categorised into less than 30min, 30min-2hours, and greater than 2hrs or don’t know	Client exit interview
Client paid for FP services	Binary (paid, not paid); whether or not clients reported they were paid for FP services in the facilities	Client exit interview
Facility relatively close to client's home	Binary (no, yes); whether or not clients reported that the facilities were the closest health facilities to their residential area	Client exit interview
***Facility-level variables***		
***Facility characteristics***		
Facility ownership	Binary (public, private); public included government and military facilities, whereas private included private for profit, private for-not-profit (faith based facilities)	Facility inventory
Facility location	Binary (urban, rural), classified based on the whether the facility is located in urban or rural areas	Facility inventory
Types of facility by level	Binary (lower, higher); lower facility included health posts, health centre, lower and medium clinics whereas higher facility included regional, zonal and referral hospitals	Facility inventory
Region	Categorical, 11 administrative regions	Facility inventory
***Structural aspects***		
Number of basic amenities	Continuous (0–6); the number of basic amenities available in the facility. It considered telephone, cell phone, computer, email, water, electricity/generator	Facility inventory
Twenty four hours staff availability	Binary (no, yes); whether or not provider available 24-hours of day (day, night, and week shifts)	Facility inventory
System to collect client opinion	Binary (no, yes); whether or not the facility has a system to collect quality assurance document such as client’s opinion sheet	Facility inventory
Availability of FP guidelines/ protocols	Binary (no, yes); whether or not the facility possessed FP guidelines/protocols on the date of survey	Facility inventory
Availability of chart/record	Binary (no, yes); whether or not the facility possessed client chart or recoding for taking notes about the clients during history taking and physical assessment	Facility inventory
Availability of supervision in the past six months	Binary (no, yes); Whether or not the facility received supervisory visit from district/regional/ zonal/federal offices in the past six months before the survey	Facility inventory
Availability of weight measurement tool	Binary (no, yes); whether or not the facility possessed weight scales	Facility inventory
Availability of Blood Pressure (BP) measurement tool	Binary (no, yes); whether or not the facility possessed BP measurement apparatus	Facility inventory
Presence of trained provider in the past 24 months	Binary (no, yes); whether or not the provider received FP related training in the past 24 months before the survey	Facility inventory
Facility has private room for counselling	Binary (no, yes); whether or not the facility’s possessed private room for counselling during FP services	Facility inventory
Number of infection prevention precaution measures	Continuous (0–14); the number of infection prevention precaution measures involved in the facility. It included availability of running water, hand washing soap, alcohol based hand rub, waste receptacle, safety box, disposable latex glove, disinfectant/antiseptics, syringe, medical masks, gowns, eye protection goggle, standard precaution guidelines, and boots.	Facility inventory
Number of FP equipment and supplies	Continuous (0–9); the number of FP equipment and supplies available in the facility. It included availability of digital BP apparatus, manual BP apparatus, stethoscope, examination light, examination bed/couch, sample FP methods, FP specific visual aids, pelvic model for IUCD demonstration, penile model for demonstrating condom use	Facility inventory
Number of quality stock organizations measures	Categorical, number of quality stock arrangements for FP commodities (No stock, 1–4, 5–8 components). It considered eight components: storage area, protected from water, protected from sunlight, protected from rodents, well ventilated, organised upon expired date, sufficient space in the stock, commodities labelled for strength and expire date.	Facility inventory
Number of days that FP services offered in a week	The median number of days that facilities providing FP services	Facility inventory
Number of contraceptive methods offered and/or prescribed	Continuous (0–12); the number of contraceptive methods offered or prescribed in the facility. The contraceptive methods included combined oral contraceptive pills, progestin only pills, progestin only injectables, male condom, female condom, implant, Intrauterine Device (IUD), periodic abstinence, emergency pills, female sterilization, and vasectomy (male sterilization), and Lactational Amenorrhea Method (LAM).	Facility inventory

FP- Family planning, IUCD- Intrauterine Contraceptive Device, BP-Blood Pressure, STI- Sexual transmitted Infections, HIV- Human Immunodeficiency Virus

#### Independent variables

The independent variables used in the analysis included client-level and facility-level factors and these are described in Table 1. The client-level variables represent characteristics related to the FP client, the process variables related to aspects of quality of care that clients encountered while receiving FP services, or during client/provider interaction. The data for the client characteristic variables were derived from the client exit interviews and the process variables were from the client-provider observations. The facility-level variables included the characteristics of the health facility and structural issues in the facility where clients received FP services. The data for these variables were derived from the facility inventory.

### Statistical analysis

Descriptive and summary statistics were used to describe the client-level and facility-level variables. All the analysis was conducted after applying client weights to adjust for disproportionate sampling and non-responses. To examine the magnitude of the relationships between different client-level and facility-level factors associated with client satisfaction, multilevel logistic regression analyses were performed. The rationale for using multilevel modelling was the following. Firstly, quality of care in FP services is influenced by the characteristics of different levels (clients, health care providers, and health facilities). Analysing variables from different levels at one single common level using the standard binary logistic regression model could create bias due to correlation between clients within the same facility. A multilevel model allows us to consider the individual level (client-level) and the facility-level in the same analysis, rather than having to choose one or the other. Secondly, the collected data had a hierarchical structure where client’s data (level 1) nested in the facility (level 2), with the likelihood that clients’ satisfaction would be correlated within the characteristics the health facilities where they received FP services.

Two-level logistic mixed-effects regression modelling was used to examine the effects of the client (level 1) and facility (level 2) variables. We ran four models: an empty model (null model)-without covariates; a model containing only client factors; a model containing only facility-level variables; and a model comprising both the client and facility-level variables.

The following equation illustrates the multilevel modelling for client satisfaction.

The full model Log [$\frac{Pij}{\left(1-Pij\right)}$] = β_0_ + β_1_Cij + β_2_Fj + μj + εij where:

i and j are the level 1 (client) and level 2 (facility) units respectively;
pij is the probability of client satisfaction occurrence in client i in a facility j that provided FP services; the β’s are the fixed effects coefficients;
C and F refer to client-level and facility-level explanatory variables, respectively;
μ shows the random effects for the jth cluster; and
ε, the random error at the client-level.

The distribution of μj was normal with mean 0 and variance σ^2^μ_0_. The Intra-Class Correlation (ICC) was calculated using between-cluster (facility) variance and within cluster (facility) variance [ICC = σμ^2^/ (σμ^2^+ π2 /3)]. The ICC was used to assess the influence of unobserved facility-level variables on client satisfaction. Univariate mixed-effects logistic regression analysis was performed to estimate the crude odds ratios at 95% confidence interval. Those variables that had p-value of less than 0.2 in this model were included in the multivariate mixed-effects logistic regression analysis.

The model fitness was assessed using Akaike Information Criterion (AIC) and Likelihood Ratio (LR) test. Variance Inflator Factor (VIF) was employed for checking multicollinearity among the independent variables. The fixed effect sizes (measure of associations) of the client and facility-level factors on client satisfaction were expressed using the Odds Ratio (OR) and 95% Confidence Interval (CI) whereas variance (measure of variations) was used to express the random effects. To account for the complex sampling strategy, weighting was employed during the analysis as well as when performing the ICC and AIC calculations. A p-value of less than 0.05 was applied used to declare level of significance. All statistical analyses were carried out with STATA 14 (Stata Corporation, College Station, TX, USA).

## Ethical approval

Ethical approvals were obtained from the Scientific and Ethical Review Committee (SERC) at the Ethiopian Public Health Institute (EPHI) and the University of Adelaide Human Research Ethics Committee (HREC).

## Results

### Description of client characteristics

The clients’ characteristics are shown in Table 2. The majority of the clients were female, aged below 35 years (83.2%), with the mean age of 28.1 years (SD = 13.2). Many had no education (44%) or only had primary education (34%). Nearly one third (30%) were new FP clients.

**Table 2:** Description of clients’ characteristics (n = 1247).

Variables	Frequency	Percent
**Sex**		
Female	1246	99.9
Male	1	0.1
**Age (in years)**		
< = 24	487	39.0
25–34	550	44.2
> = 35	206	16.5
Missing	3	0.3
**Educational status**		
No education	552	44.2
Primary	428	34.4
Secondary	189	15.1
Tertiary	74	6.0
Missing	3	0.3
**Types of client**		
New client	372	29.9
Repeated client	871	69.9
Missing	2	0.2

### Client satisfaction and process aspects in the provision of family planning services

Table 3 shows level of client satisfaction and various process aspects of quality of care in the provisions of family planning services in Ethiopia. Of 1247 clients who had a client-provider observation and participated in an exit interviews, 729 (59%) were more satisfied with FP services. In terms of information provision to the clients, nearly three quarters (73%) were informed about how to use the contraceptive method and nearly half (46%) about potential negative side effects. While only 655 (55%) were informed on what to do if a problem occurs, most (93%) were informed about when to return for follow-up. Very few clients were asked about their partner’s attitude towards FP method (10%), their number of sexual partners (3%), or their HIV/STI perceived risk (8%). With regard to client’s concern about the contraceptive methods, 541 (43%) of them left without their concerns being addressed. Only two percent of clients were provided information about condom use for Sexual Transmitted Infection (STI) prevention and dual contraceptive method use for Human Immunodeficiency Virus (HIV) and/or STI prevention. Privacy was not maintained for 460 (37%) clients and confidentiality was not assured for the majority (88%) of clients. The median duration of the client consultation was 10 minutes of which more than half (52%) were consulted for less than 10 minutes. The average number of history and physical assessments was 3.2 (SD = 2.3). In this regard, 187 (15%) of clients had not been assessed through neither history taking nor physical examination. The overwhelming majority of clients (89%) were not required to pay for the service and 59% waited less than 30 minutes before receiving FP services (Table 3).

**Table 3:** Client satisfaction and process aspects during client-provider interaction (n = 1247).

Variables	Frequency	Percent
**Client satisfaction**		
Less satisfied	518	41.5
More satisfied	729	58.5
**Information provided about how to use the contraceptive method**		
No	319	26.7
Yes	877	73.3
**Information provided about the contraceptive method’s potential side effects**		
No	549	46.4
Yes	635	53.6
**Information provided on what to do if a problem occurs**		
No	538	45.1
Yes	655	54.9
**Information provided on when to return for follow-up**		
No	83	6.9
Yes	1,116	93.1
**Partner’s attitude towards FP asked**		
No	1,123	90.10
Yes	124	9.90
**Client’s number of sexual partner asked**		
No	1,204	96.60
Yes	43	3.40
**Client's perceived risk of HIV/STI asked**		
No	1,145	91.80
Yes	102	8.20
**Client’s concerns about the methods addressed**		
No	541	43.40
Yes	706	56.60
**Information about condom use for STI prevention provided**		
No	1,221	97.90
Yes	26	2.10
**Information about dual method use for HIV/STI prevention provided**		
No	1,228	98.50
Yes	19	1.50
**Clients asked question to the providers**		
No	700	56.10
Yes	547	43.90
***Privacy and confidentiality issues***		
**Auditory privacy maintained**		
No	319	25.60
Yes	928	74.40
**Visual privacy maintained**		
No	440	35.30
Yes	807	64.70
**Both auditory and visual privacy maintained**		
No	460	36.90
Yes	787	63.10
**Confidentiality assured**		
No	1,100	88.20
Yes	147	11.80
**Duration of consultation in minutes (median = 10min)**		
< 10 minutes	651	52.2
10–15 minutes	379	30.4
>15 minutes	211	17.0
Missing	5	0.4
**Number of history and physical assessments**		
0	187	15.00
1–2	363	29.20
3–6	561	45.00
7–12	191	10.80
>13	0	0
**Client’s waiting time**		
<30min	738	59.2
30min-2hrs	246	19.7
>2hrs	124	10.0
Don’t know	138	11.1
**Client paid for FP services**		
No	1,103	88.5
Yes	144	11.5
**Facility relatively close to client's home**		
No	240	19.3
Yes	1,005	80.6
Missing	1	0.1

FP- family planning, STI- Sexual Transmitted Infections, HIV- Human Immunodeficiency Virus

### Description of the characteristics and structural aspects of health facilities

**Fig 2:**
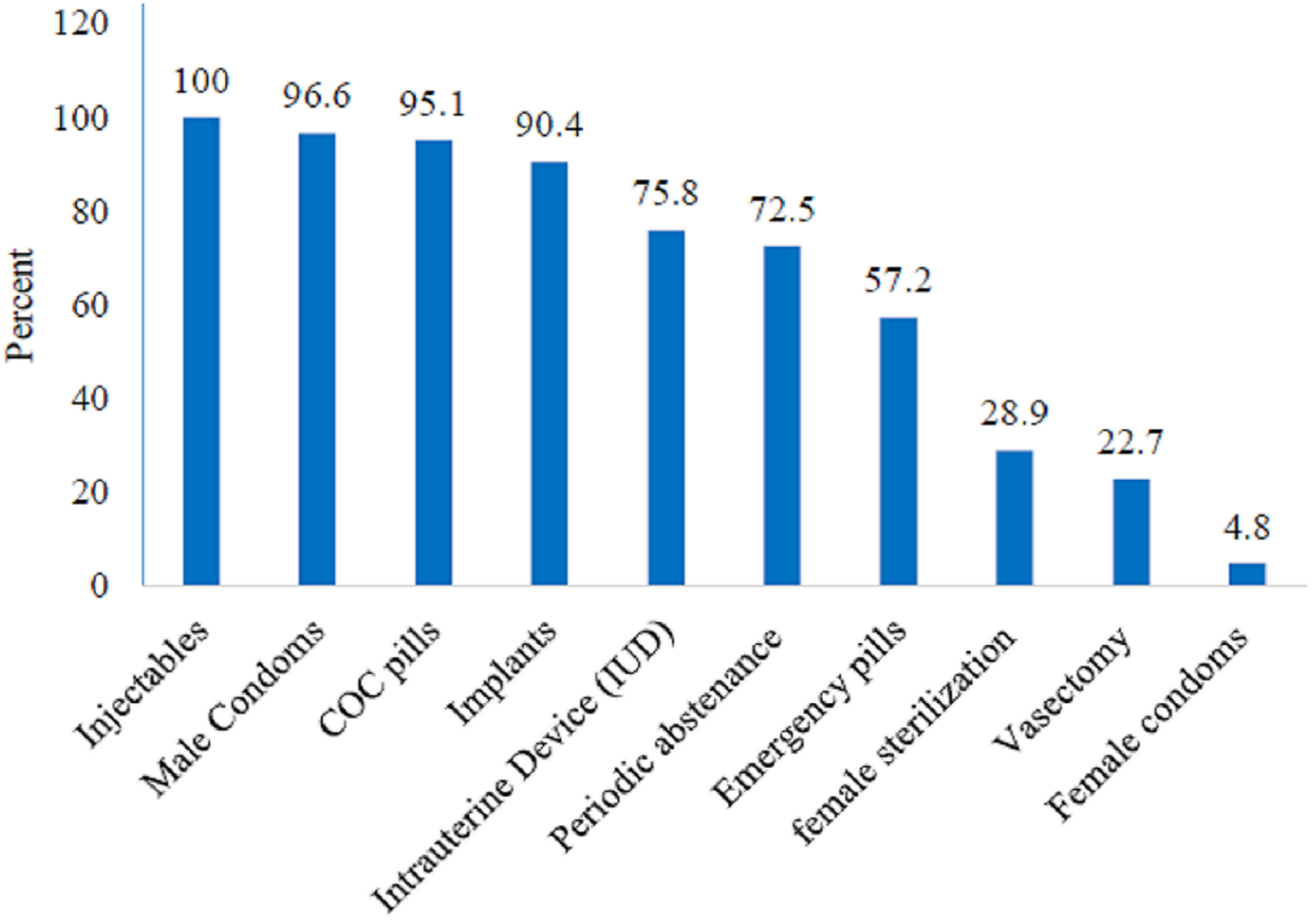
Percentage of facilities by type of available family planning methods during the survey (n = 374).

Table 4 shows the characteristics of health facilities and their structural aspects. Of the 374 facilities where client-provider observations were conducted, 350 (94%) were publicly owned, just over half (53%) were located in urban areas and about 1 in 5 facilities (17%) were higher level facilities. Nearly three-quarters (72%) of health facilities were from Oromia, Amhara, Southern Nations, Nationality and People Region (SNNPR), and Tigray regions. Two-thirds of facilities (66%) had 1–3 basic amenities (median = 3.2). More than a quarter of facilities (27%) did not have staff available for 24 hours, and 75% did not have systems to collect client’s opinion (i.e client’s opinion sheet) one year prior to the survey. About 4 in 10 of facilities (43%) did not have FP guidelines/protocols to guide the FP practice. Client’s chart/record were available in over two-thirds (69%) of facilities. Health facility supervision is conducted as part of the facilities’ monitoring and evaluation system, and aims to provide technical support for staff and identify the facilities’ demand for equipment and materials. In this regard, only two-thirds (66%) of the facilities reported receiving supervision from district/zonal/regional/federal health offices in the six months prior to the survey. The majority of facilities did not have weight and blood pressure measurement tools, 77% and 79% of facilities respectively. In 68% of the facilities, health providers received FP related training in the 24 months before the survey. Most (91%) facilities possessed a private room for counselling during FP services (Table 4). While injectable contraceptive methods were provided and/or prescribed in all facilities, female condoms were provided and/or prescribed in less than five percent of the facilities (**Fig** 2).

**Table 4:** Description of the characteristics and structural aspects of health facilities (n = 374).

Variable	Frequency	Percent
**Facility ownership**		
Private	24	6.5
Public	350	93.5
**Facility location**		
Rural	198	52.9
Urban	176	47.1
**Type of facility by level**		
Lower-level facility	311	82.9
Higher-level facility	64	17.1
**Region**		
Tigray	53	13.8
Afar	6	2.1
Amhara	80	20.9
Oromia	84	21.9
Somalie	8	2.1
Benshangul-Gumuz	23	6.0
SNNPR	58	15.1
Gambella	5	1.3
Harari	14	3.7
Addis Ababa	34	8.9
Dire Dawa	16	4.2
**Number of basic amenities (median = 3.2)**		
0	7	1.9
1–3	248	66.3
4–6	119	31.8
**Twenty four hours staff availability**		
Not available	101	27.1
Available	273	72.9
**System to collect client opinion**		
No	280	74.8
Yes	94	25.2
**Availability of FP guidelines/protocols**		
No	160	42.7
Yes	215	57.3
**Availability of chart/record**		
No	115	30.7
Yes	259	69.3
**Availability of supervision in the past six months**		
No	128	34.2
Yes	246	65.8
**Availability of weight measurement tool**		
No	297	79.4
Yes	77	20.6
**Availability of BP measurement tool**		
Not observed	290	77.4
Observe	85	22.6
**Presence of trained provider in the facility in the last 24 months**		
No	121	32.3
Yes	254	67.7
**Facility has private for counselling**		
No	32	8.6
Yes	342	91.4
**Number of IP precaution measures**		
1–5	65	17.5
6–10	248	66.1
11–14	71	16.4
**Number of FP equipment’s and supplies**		
1–3	55	14.8
4–6	236	62.9
7–9	83	22.3
**Number of quality of stock organization measures**		
No stock	190	50.7
1–4 stock component	39.	10.7
5–8 stock component	145	38.6
**Number of days that FP services offered in a week (median n = 5.4)**		
**Number of contraceptive methods offered and/or prescribed**		
1–3 methods	28	7.4
4–6 methods	132	35.4
7–11 methods	214	57.2

*IP- Infection Precaution, FP- Family Planning, SNNPR-Southern Nations, Nationality and People Region

### Determinants of quality of care in family planning services

The fixed effects (measure of association) and random effects (measure of variation) for client satisfaction are presented in Table 5. The results of the empty model (model I) showed a significant variability in the odds of client satisfaction between health facilities (variance = 1.61). Similarly, the Intraclass Correlation Coefficient (ICC) estimated in the null model (model I) showed that one third (32.8%) of the differences in the client satisfaction can be attributed to the health facilities where clients received FP services. In model II, only client-level variables were included. In this model, client age, client’s waiting time, number of history and physical assessments, information provided about the contraceptive method’s potential side effects, and duration of consultation were found significant. Model III where only facility-level variables included, facility location, availability of supervision in the past six months, and availability of FP guidelines/protocols were found significant. In Model IV (full model), after adjusting for client and facility-level factors, client age, client’s waiting time, information provided about the contraceptive method’s potential side effects, number of history and physical assessments, being an urban facility, and availability of FP guidelines/protocols were the factors that had a statistically significant association with client satisfaction.

**Table 5:** Multivariate mixed-effects logistic regression analysis to identify the client and facility-level determinants of quality of care in family planning services in Ethiopia (n = 1247).

Variables	Univariate model COR (95% CI)	Model I (empty) AOR (95% CI)	Model II AOR (95% CI)	Model III AOR (95% CI)	Model IV AOR (95% CI)
***Client-level variables***					
**Client age**	1.02 (1.00–1.04)*		1.03 (1.00–1.05)*		1.02 (1.00–1.04)*
**Client’s waiting time**					
<30min	**1**		1		1
30min- 2hrs	0.19(0.07–0.53)**		0.10 (0.03–0.31)**		0.11(0.03–0.33)*
>2hrs or don’t know	0.84 (0.26–2.68)		0.41 (0.09–1.85)		0.47(0.11–2.08)
**Privacy maintained**					
No	1		1		1
Yes	1.68 (0.67–4.25)		1.78 (0.51–6.20)		1.96(0.57–6.72)
**Number of history and physical assessments**	1.15 (1.01–1.31)*		1.20 (1.03, 1.40)*		1.19(1.03–1.34)*
**Information provided about the contraceptive method’s potential side effects**					
No	1		1		1
Yes	**4.19 (1.80–9.72)**		5.63 (2.14–14.82))**		5.22(2.13–12.80)**
**Duration of consultation**	**0.93 (0.85–1.02)**		0.90 (0.82–0.99)*		0.90 (0.82–0.99)
***Facility-level variables***					
**Facility ownership**					
Private	1			1	1
Public	8.43 (1.41–50.31)			4.32 (0.63–29.70)	5.39 (0.69–41.75)
**Types of facility by level**					
Lower-level	1			1	1
Higher-level	**0.27 (0.10–0.71)**			0.39 (0.14–1.07)	0.86 (0.26–2.83)
**Facility by location**					
Rural	1			1	1
Urban	1.50 (0.41–5.55)			3.54(1.00–12.50)*	4.61(1.04–20.58)*
**Number of basic amenities**	0.65 (0.38–1.12)			0.68 (0.41–1.15)	0.43 (0.22–0.84)
**Availability of supervision in the past six months**					
No	1			1	1
Yes	4.35 (1.25–15.07)*			2.98 (1.18–7.54)*	2.54 (0.81–7.97)
**Number of quality stock organization measures**					
No stock	**1**			1	1
1–4 stock	0.77 (0.16–3.86)			1.23 (0.35–4.42)	0.46 (0.10–2.00)
5–8 stock	1.04 (0.31–3.46)			1.41 (0.57–3.54)	1.30 (0.52–1.24)
**System to collect client opinion**					
No	**1**			1	**1**
Yes	2.25 (0.46–11.07)			1.53 (0.54–4.36)	1.36 (0.35–5.26)
**Contraceptives offered/ prescribed**	0.77 (0.53–1.09)			0.85 (.62–1.17)	0.80 (0.52–1.24)
**Availability of FP guidelines/protocols**					
No	1			**1**	**1**
Yes	3.39 (1.45–11.07)*			3.62 (1.23–10.64)*	4.90 (1.19–20.19)*
**Number of IP precaution measures**	0.84 (0.66–1.07)			0.93 (0.72–1.19)	0.90 (0.66–1.22)
**Number of FP equipment and supplies**	0.86 (0.66–1.12)			1.00(0.83–1.20)	0.96 (0.77–1.21)
***Random effect***					
**Variance (SE)**		1.61 (0.34)*	9.38 (4.91)*	3.49 (1.12)*	6.15 (2.35)*
**ICC (%)**		32.8%	34.4%	28.1%	31.5%
**Model Fitness**					
**Log Likelihood**		-768.13	-674.6	-754.60	-664.52
AIC		1540.3	1367.3	1539.2	1372.05

*P-value< 0.05

**p-value<0.01

AOR- Crude Odds Ratio, AOR- Adjusted Odds Ratio, CI- Confidence Interval, IP- Infection Prevention FP- Family Planning, SE-Standard Error, ICC- Intraclass Correlation Coefficient, AIC- Akaike Information Criterion

The odds of being more satisfied were lower by 89% for clients who waited 30 minutes to two hours compared to clients who waited less than 30 minutes before being seen by the provider for consultation in FP services (AOR = 0.11, 95% CI: 0.03–0.33). Clients who were informed about the potential side effects of the contraceptive methods had about five times higher odds of being more satisfied than those who were not provided with information about potential side effects (AOR = 5.22, 95% CI: 2.13–12.80). The odds of being more satisfied were increased by 19% for each unit increase in number of history and physical assessments (AOR = 1.19, 95% CI: 1.03–1.34). There was borderline significance association between age of the client and client satisfaction (OR = 1.02; 95% CI: 1.00, 1.04, p-value = 0.049).

Similarly, those clients who received FP services in urban facilities had about five times higher odds of being more satisfied than those rural facilities. The availability of FP guidelines/protocols was also a significant factor in determining client satisfaction. In this regard, the odds of clients being more satisfied were nearly five times higher in facilities with FP guidelines/protocols as compared to those facilities without FP guidelines/protocols (AOR = 4.90, 95% CI: 1.19–20.19).

## Discussion

Using data from the Ethiopian Services Assessment Plus (ESPA+) survey 2014 data, this study assessed the client-level and facility-level determinants of quality of care in FP services in Ethiopia. After controlling the effects of confounders, the findings demonstrate that both client-level and facility-level factors were associated with quality of care in FP services in Ethiopia. At the client-level; age, client’s waiting time, information provision about the potential side effects of the contraceptive methods, and number of history and physical assessments were significantly associated with client satisfaction in FP services. These client-level factors are mostly related to the process of FP services provision. At the facility-level; the availability of FP guidelines/protocols and facility location were significant factors. Excluding client’s age and facility location, the factors identified in the present study reaffirmed four out of the six aspects of the quality of care identified in Bruce/Jain framework [26]. However, unlike this framework, having higher number of contraceptive methods in the health facility (a proxy indicator for the contraceptive method mix) and information on when to return for follow-up were not significant in the present study.

A recent systematic review of studies undertaken in African countries to identify factors determining quality of care in FP services suggests that client waiting time is an important factor associated with quality of care in FP services [32]. Similarly, the present study showed significant association between client’s waiting time and client satisfaction. The odds of being more satisfied in FP services was lower for clients who waited between 30 minutes to two hours as compared to those clients waited less than 30 minutes. When taking the median consultation time of 10 minutes into consideration, the findings that nearly one-thirds of clients waited for more than 30 minutes may suggest either the facilities had too few FP providers relative to the number of clients, or/and too short service opening hours.

The other key client-level factor was the provision of information about the potential side effects of the contraceptive methods. There was higher odds of being more satisfied in FP services when the clients provided information about the potential side effects contraceptive methods. This is in agreement with a previous study conducted in Finland that underscored provision of information about potential side effects of certain contraceptive methods improved user satisfaction [40]. Additionally, higher number of history and physical assessments for clients’ was associated with greater odds of being more satisfied with FP services. In this study, however, about 1 in 7 clients (15%) neither had history nor a physical examination taken. A study conducted in Kenya [41] showed similar finding. These results inform about the need for providers to be retrained about, and supervised for, making sure that they examine the clients and effectively communicate information about the potential side effects.

Besides the client-level factors, the availability of FP guidelines/protocols was an important facility-level structural factor associated with quality of care in FP services. The odds of being more satisfied with FP services was higher for facilities that possessed FP guidelines/protocols as compared to facilities that did not. The availability of guidelines/protocols in the facility suggests that health providers in those facilities have the opportunity to gain up-to-date knowledge about the FP methods which include the potential side effects of different contraceptive methods and the counselling approach. This would in turn help provider to offer client-tailored counselling during FP services. Previous study conducted in Jordan showed similar findings in that provision of protocols to guide client’s counselling have improved clients satisfaction with the FP services [42].

Another important determinant of client satisfaction in FP services was the location of the health facility. This study found that those clients who received FP services in urban facilities had higher odds of being more satisfied than rural facilities. This difference may be explained by the following reasons. Firstly, FP clients of rural facilities are likely rural residents who may have less information about the contraceptive methods due to less access for media. Secondly, rural facilities may have a shortage of experienced FP providers due to the possibility that experienced providers would transfer to urban facilities while newly graduated health providers assigned in rural facilities. Thirdly, some contraceptive methods such as female sterilization and Intrauterine Device (IUD) might be available only in urban facilities. As a result, client choice of contraceptive methods may not be fulfilled and eventually they could remain less satisfied with the services.

## Strength and limitations

A strength of this study is that it is the first ever study that provides an assessment of the quality of care in FP services in Ethiopia and to identify the determinants of quality of care in FP services based on a nationally representative sample. Secondly, this study provides findings that can be used as a baseline for future study including future rounds of ESPA+ surveys and also for assessing the impact of policy and strategies to improve FP services in Ethiopia.

However, there are some limitations. Given that characteristics of health providers can influence the quality of care in FP services, the ESPA+ 2014 data involved a random sample of health providers rather than interviewing providers involved in FP services provision. A first limitation of the study is that it was not possible to control provider related confounders. A second limitation is that some factors that may be important in determining quality of FP services, including marital status, religion, and residence were not included in the analysis as this information was not collected in the ESPA+ 2014 survey. Thirdly, collecting the observation data during client-provider interaction, might have produced social desirability bias in that health providers may have altered their behaviour, and performed better, due to them being aware that their interactions were being evaluated. This may also apply to client responses.

## Policy implications

Our results have a number of policy implications. The provision of information for clients including the contraceptive method’s potential side effects is necessary and so future policies should target in the dissemination and improving uptake of best practice guidelines for providers working in the FP services. In addition, there is a need to improve health providers’ skills in terms of conducting thorough history taking and physical assessment for clients attending FP services and this could be done through targeted training. Appropriate strategies including making proper arrangement for long service hours and deploying trained providers from other reproductive health or maternity services are important to minimize clients waiting time in health facilities. The difference between rural and urban FP services need to be addressed and may a number of policies that focus on improving services in rural areas such as recruitment of health providers, provision of resources and education programs for health providers and clients.

## Conclusion

Quality of care in FP services in Ethiopia is influenced by client-level and facility-level factors. Shorter waiting times, the provision of information about the contraceptive method’s potential side effects, improved history and physical assessments, the availability of FP guidelines/protocols, and being urban facilities were important determinants of quality of care in FP services in Ethiopia.

## References

[1] WHO U, UNFPA, The World Bank, UN Population Division (2015) Trends in maternal mortality: 1990 to 2015. Geneva, Switzerland: WHO, UNICEF, UNFPA, The World Bank, UN Population Division.

[2] CSA [Ethiopia] and ICF (2016) Ethiopia Demographic and Health Survey 2016: Key Indicators Report. Addis Ababa, Ethiopia, and Rockville, Maryland, USA.

[3] WHO (1996) Mother–baby package: implementing safe motherhood in countries. World Health Organization: Geneva.

[4] UNFPA (1994) Programme of action adapted at the International Conference of Population and Developement Cairo: UNFPA.

[5] Ahmed S, Li Q, Liu L, Tsui AO (2012) Maternal deaths averted by contraceptive use: an analysis of 172 countries. Lancet 380: 111–125. doi: 10.1016/S0140-6736(12)60478-4 22784531 10.1016/S0140-6736(12)60478-4

[6] Cleland J, Conde-Agudelo A, Peterson H, Ross J, Tsui A (2012) Contraception and health. The Lancet 380: 149–156. 10.1016/S0140-6736(12)60609-6 22784533

[7] Tsui AO, McDonald-Mosley R, Burke AE (2010) Family Planning and the Burden of Unintended Pregnancies. Epidemiologic Reviews 32: 152–174. doi: 10.1093/epirev/mxq012 20570955 10.1093/epirev/mxq012 PMC3115338

[8] Collumbien M, Gerressu M, C J. (2004) Non-use and use of ineffective methods of contraception In: Ezzati M, Lopez AD, Rogers A, Murray CJL, editors. Comparative quantification of health risks: Global and regional burden of disease attributable to selected major risk factors. Geneva: World Health Organisation pp. 1255–1320.

[9] Rutstein SO (2005) Effects of preceding birth intervals on neonatal, infant and under-five years mortality and nutritional status in developing countries: evidence from the demographic and health surveys. Int J Gynaecol Obstet 89 Suppl 1: S7–24. 15820369 10.1016/j.ijgo.2004.11.012

[10] Conde-Agudelo A, Belizan JM, Norton MH, Rosas-Bermudez A (2005) Effect of the interpregnancy interval on perinatal outcomes in Latin America. Obstet Gynecol 106: 359–366. doi: 10.1097/01.AOG.0000171118.79529.a3 16055588 10.1097/01.AOG.0000171118.79529.a3

[11] FMOH (2011) National Guideline for Family Planning Services in Ethiopia. Addis Ababa: FMOH Ethiopia,.

[12] USAID/Africa Bureau (2011) Three Successful Sub-Saharan Africa Family Planning Programs: Lessons for Meeting the MDGs.

[13] CSA [Ethiopia] and ORC Macro (2001) Ethiopia Demographic and Health Survey 2000. Addis Ababa, Ethiopia and Calverton, Maryland, USA: Central Statistical Authority [CSA] and ORC Macro.

[14] CSA [Ethiopia] and ORC Macro (2006) Ethiopia Demographic and Health Survey 2005 Addis Ababa, Ethiopia and Calverton, Maryland, USA:.

[15] CSA [Ethiopia] and ICF International (2012) Ethiopian Demographic and Health Survey report 2011. Addis Ababa, Ethiopia and Calverton, Maryland, USA: Central Statistical Agency and ICF Internationa.

[16] Blanc A, Curtis S, Croft T (2002) Monitoring contraceptive continuation: links to fertility outcomes and quality of care. Stud Fam Plann 33: 127–140. 12132634 10.1111/j.1728-4465.2002.00127.x

[17] Askew I, Mensch B, Adewuyi A (1994) Indicators for Measuring the Quality of Family Planning Services in Nigeria. Studies in Family Planning 25: 268–283. 7871552

[18] Kaufman J, Zhang Z, Qiao X, Zhang Y (1992) The quality of family planning services in rural China. Stud Fam Plann 23: 73–84. 1604461

[19] Koenig MA, Hossain MB, Whittaker M (1997) The Influence of Quality of Care upon Contraceptive Use in Rural Bangladesh. Stud Fam Plann 28: 278–289. 9431649

[20] RamaRao S, Mohanam R (2003) The quality of family planning programs: concepts, measurements, interventions, and effects. Stud Fam Plann 34: 227–248. 14758606 10.1111/j.1728-4465.2003.00227.x

[21] Ali M (2001) Quality of care and contraceptive pill discontinuation in rural Egypt. Journal of Biosocial Science 33: 161–172. 11284624 10.1017/s0021932001001614

[22] Sanogo D, RamaRao S, Jones H, N'Diaye P, M'Bow B, Diop CB. (2003) Improving Quality of Care and Use of Contraceptives in Senegal. Afr J Reprod Health 7: 57–73. 14677301

[23] Birhane K, Hagos S, Fantahun M (2015) Early discontinuation of implanon and its associated factors among women who ever used implanon in Ofla District, Tigray, Northern Ethiopia. International Journal of Pharma Sciences and Research 6(3): 544–551.

[24] FMOH (2015) Health Sector Transformation Plan (HSTP) 2015/16–2019/20 (2008–2012 EFY). Addis Ababa, Ethiopia FMOH

[25] Donabedian A (1988) The quality of care: How can it be assessed? JAMA 260: 1743–1748. 3045356 10.1001/jama.260.12.1743

[26] Bruce J (1990) Fundamental elements of quality of care: A simple framework. Studies in Family Planning 21: 61–69. 2191476

[27] Fantahun M (2005) Quality of family planning services in Northwest Ethiopia. Ethiopia J Health Dev 19: 195–200.

[28] Kebede Y (2007) Quality of family planning service in Dembia district, north west Ethiopia. Ethiopian medical journal 45: 29–38. 17642155

[29] Loha E, Asefa M, Jira C, Tessema F (2003) Assessment of quality of care in family planning services in Jimma Zone, Southwest Ethiopia. [Ethiop J Health Dev 18: 8–18.

[30] Tafese F, Woldie M, Megerssa B (2013) Quality of family planning services in primary health centers of Jimma Zone, Southwest Ethiopia. Ethiopian journal of health sciences 23: 245–254. 24307824 10.4314/ejhs.v23i3.7 PMC3847534

[31] Argago TG, Hajito KW, Kitila SB (2015) Client’s satisfaction with family planning services and associated factors among family planning users in Hossana Town Public Health Facilities, South Ethiopia: Facility-based cross-sectional study International Journal of Nursing and Midwifery 7: 74–83.

[32] Tessema GA, Streak Gomersall J, Mahmood MA, Laurence CO (2016) Factors Determining Quality of Care in Family Planning Services in Africa: A Systematic Review of Mixed Evidence. PLoS ONE 11: e0165627 doi: 10.1371/journal.pone.0165627 27812124 10.1371/journal.pone.0165627 PMC5094662

[33] EPHI FMOH, ICF International, (2014) Key findings on Ethiopia Service Provision Assessment Plus (ESPA+) Survey 2014. Addis Ababa, Ethiopia: Ethiopian Public Health Institute, Federal Ministry of Health, ICF International,.

[34] PRB (2016) 2016 Wordl Population Data Sheet with a Special Focus on Human Needs and Sustainable Resources Population Reference Bureau (PRB).

[35] FMOH (2014) Ethiopia’s Fifth National Health Accounts 2010/2011. Addis Ababa, Ethiopia: FMOH.

[36] The DHS Program (2014) The Service Provision Assessment (SPA).

[37] Hameed W, Azmat SK, Ali M, Hussain W, Mustafa G, Ishaque M, et al (2015) Determinants of Method Switching among Social Franchise Clients Who Discontinued the Use of Intrauterine Contraceptive Device. Int J Reprod Med 2015: 941708 doi: 10.1155/2015/941708 26576454 10.1155/2015/941708 PMC4630392

[38] Strobino DM, Koenig M, Grason HA (2000) Approaches and Indicators for Measuring Quality in Region VIII Family Planning Programming. Baltimore, MD.

[39] Kolenikov S, Angeles G (2004) The Use of Discrete Data in Principal Component Analysis with Applications to Socio-Economic Indices. CPC/MEASURE Working Paper. Chapel Hill, USA.

[40] Backman T, Huhtala S, Luoto R, Tuominen J, Rauramo I, Koskenvuo M (2002) Advance information improves user satisfaction with the levonorgestrel intrauterine system. Obstetrics & Gynecology 99: 608–613. 12039121 10.1016/s0029-7844(01)01764-1

[41] Agha S, Do M (2009) The quality of family planning services and client satisfaction in the public and private sectors in Kenya. International Journal for Quality in Health Care 21: 87–96. doi: 10.1093/intqhc/mzp002 19190135 10.1093/intqhc/mzp002

[42] Kamhawi S, Underwood C, Murad H, Jabre B (2013) Client-centered counseling improves client satisfaction with family planning visits: evidence from Irbid, Jordan. Glob Health Sci Pract 1: 180–192. doi: 10.9745/GHSP-D-12-00051 25276531 10.9745/GHSP-D-12-00051 PMC4168569

